# The persistent educational digital divide and its impact on societal inequality

**DOI:** 10.1371/journal.pone.0286795

**Published:** 2024-04-03

**Authors:** Glenn L. Pierce, Paul F. Cleary

**Affiliations:** 1 Institute of Security and Public Policy School of Criminology and Criminal Justice, Northeastern University, Boston, Massachusetts, United States of America; 2 School of Management, University of Massachusetts-Boston, Boston, Massachusetts, United States of America; Universidade Federal de Minas Gerais, BRAZIL

## Abstract

Computers and the Internet are widely recognized as fundamental to academic and future success on both the individual and the societal level. Moreover, the academic success of school-age children is now increasingly tied to access to educational technology, a reality that became even more apparent during the pandemic. While academic performance is viewed as the major outcome of using educational technology, this study looks at a crucial early stage in the educational technology value chain, specifically; 1) to what extent do students use computers and the Internet in their homes and at school and 2) what is the extent and nature of disparities in student access to educational technology. This study was conducted using the national CPS 2019 Computer and Internet Use Survey of 23,064 school age children. We used bivariate tables and multivariate logistic regression analysis to analyze the data. Results indicate that substantial disparities in the use of educational technology exist in the U.S. Overall, 28.0% of school age children reported they did not use the Internet at school or at home and another 22.8% reported using the Internet at home but not at school. Significantly, individual and community demographic characteristics and household and school technology resources contribute to these disparities. It is clear that if fundamental educational technology and the resources needed to effectively achieve academic success are unavailable in the home, then they must be provided in schools. Without educational technology and resources, the societal value added through growing use of this technology will not materialize for our students. We conclude that committing to increasing educational technology resources in the schools will have multiple future societal benefits and improve the effectiveness of the educational technology value chain.

## Introduction

Computers and the Internet are widely recognized as central to modern communication and crucial to global economic competitiveness. Equally important, access to computing and the Internet is considered fundamental to success in adding value on both the individual and the societal level. Also, it is widely understood that not everyone in the U.S. has equal access to this technology and that disparities in access and use exist. Public policy responses to recognized societal computer and Internet gaps range from viewing access as a societal right to ad hoc responses, which ignore the larger picture. As [1] pointed out twenty years ago, access to public and private sector institutions in a democratic society, as well as adding value and productivity, requires access to computers and the Internet, and this is even truer today. What has been less clear is the extent of this disparity in society. In this research, we examine empirically, the nature and extent to which disparities in computer and Internet access exist in K-12 education and the impact that this missing initial link in the educational technology value chain can have on the ultimate success of a student’s educational objectives.

### Systems view and educational technology value chains

The origins of the overall approach taken in this paper are found in general system theory [2] and in value chain theory [3]. A system can be identified as a complex entity of interacting and interrelated elements that are open to, and interact with, their appropriate environments. The systems view looks at the world through relationships and integration. Systems are integrated or connected into a whole entity whose properties cannot be reduced to those of smaller units. Instead of concentrating on basic building block elements, the systems approach emphasizes the organization of elements into a processed whole [2].

An important aspect of systems is their dynamic and changing nature. Their forms are not rigid structures but are flexible yet stable outcomes of underlying processes. Systems thinking is process thinking and form becomes associated with process [2]. In this application, the “process” is providing education to students through the support and use of educational technology through a series of interrelated links that form an educational technology value chain and result in academic achievement.

Value chain systems provide the theoretical core of how to view the structure, dynamics and impacts that a lack of access to computing and Internet resources have on students. Porter focused on business applications and defined a value chain as a collection of interrelated activities that are performed by a business to create value for its customers [3, 4]. A value chain is viewed as an analytic construct that identifies how each production stage in a business process impacts the overall creation of the product or service that enhances its features, quality and general market appeal.

Value chains enhance internal product or service value at each process stage which leads to higher quality and often less expensive products and services. Conducting a value chain analysis involves implementing improvements and reducing costs at each interrelated process stage, which can lead to overall added value, which potentially results in higher profitability and increased competitive advantage for an organization [3].

Value chain theory has been adapted to several applications in areas other than private sector business including: intellectual capital in education through a value chain perspective, assessing intangible resources needed for educational process quality [5, 6]; higher education academics and administrative institution applications [7, 8], and use of value chains in delivery of distance learning educational services [9].

In the educational environment, the applications of value chains (educational supply chains) can enhance the clarity and efficiency of product and service delivery in various educational and learning setting, however, it is clear that no one component or link in the educational technology value chain will be able to assist in closing educational disparities, particularly at the K-12 level by itself. As part of an educational delivery system, these constituent components must work together in order to assist in providing educational value [2].

In this paper, we adopted Porter’s value chain approach to applications and value added outcomes in educational technology [10]. These technologies are part of an educational value chain system that extents from the availability of technology in households to the ability of school systems to integrate and deliver educational technology into educational curricula and environments. The focus of this current investigation for use in the education sector, is on factors that affect the critical first stage (component) in the educational technology value chain system, specifically, access to a computer and the Internet.

### Use of computing resources, the internet and expertise

Despite the importance and need of computer and Internet technology for individuals and society the rate of growth of household Internet access and use in the U.S. falls short of this need. Today, although Internet penetration has continued to grow in the United States, it continues to grow unevenly. There is extensive evidence of persistent digital disparities in computing technology and Internet use [11]. The current household penetration rate is still estimated as somewhere between 74–86% [11, 12]. This observed disparity in Internet use can be attributed to persistent obstacles that prevent many from gaining use [13] and consequently, a substantial digital divide persists [11, 12, 14]. A key result of this is that many school age children in households without access, are prevented from acquiring and achieving the crucial first stage in the educational technology value chain.

The most visible indication of the digital divide has been observed during the ongoing pandemic. Those most severely affected by in person restrictions, isolation and the lack of educational resources are children engaged in educational activities at the formative K-12 level. These disparities can appear in the form of access to computing equipment, the Internet and to the knowledge, expertise and supervision required to help most children optimize the use of these educational technology resources.

Since today’s economy is increasingly based on computer control, information and electronic communication, it is critical that all students in the K-12 system have access to this technology early on. The fast-changing nature of work in today’s economy reflects the continuing evolution and importance of computer technology, automation and support. Consequently, mastering skills in educational/digital technology has become more important than ever. In order to retain a preeminent technological and economic position in the world, the U.S. must improve access to and facilitate delivery and use of computing technology to children in the K-12 educational system. This study focuses on factors affecting the access of school-age children to computers and the Internet at home and at school.

### Use of computing resources, the internet and expertise—Post pandemic

Although considered significant prior to the pandemic, it has now become even more evident that access to and use of computing resources is a necessity for K-12 education. The potential impact of digital disparities on academic achievement has been well documented [15, 16]. Educators have concluded that the presence of computing resources, the Internet and household expertise can have multiple benefits on the educational achievement of school-age children, not the least of which are improvements in STEM performance, since school curricula now routinely require computer use and support and are central to educational content and delivery.

In addition, an important consequence of the pandemic is that it has highlighted and exacerbated the already persistent gap between the “haves” and the “have nots” in society, and particularly in education. The crisis has significantly raised the educational stakes for those without Internet access, computing resources and expertise. The events of the past year have dramatically altered how education is delivered and have significantly highlighted the growing gaps in educational technology in the delivery of academic services [17].

Another critical reason to study at home computer and Internet use and at school Internet use is that prior to the pandemic, evidence strongly indicated that educational technology significantly impacts academic performance in society [18, 19] and others have demonstrated that introducing educational technology into K-12 curricula can improve student performance [20].

Even with access to home computing resources and the Internet, there is no guarantee that computer expertise will be transferred to children successfully or consistently or that use will focus on educational activities. Reports have indicated that parental management is difficult as children spend too much time on non-educational activities such as social media or video games [21]. Potential barriers to educational access and use are numerous and include: household financial and educational constraints, student unwillingness to engage in online learning activities and parental inability or unwillingness to assist in student learning. Breaking down these barriers to remote educational use of educational technology for all is crucial; failing to do so could potentially exacerbate current disparities in educational performance. The present analysis examines these issues and potential disparities.

## Key study objectives

- Examine what is the overall use of computer and Internet technology by school-age children.
- Examine to what extent child demographics, family social and economic circumstances, and family technology resources of household adults are associated with children’s use of computers at home and the Internet at home and at school.
- Finally, examine the unique contributions of child demographics, family circumstances and technology resources to children’s use of computer and Internet technology.

## Methodologic approach

This study draws on data from the November 2019 Current Population Survey (CPS) to examine computer and Internet use among school-age children. The current research expands beyond previous studies of school-age children, by incorporating additional demographic factors not examined in previous research (i.e., citizenship status), and factors related to computing expertise and resources in online learning environments whose importance has grown during the current pandemic. The data allows us to examine how computer and Internet use by school-age children varies by the characteristics of individual children, the socioeconomic characteristics of their households (families), and the availability of household computing resources and household technology expertise.

### Sources of data

The CPS survey is administered by the United States Bureau of the Census on a monthly basis. In the November 2019 CPS survey, approximately 47,000 randomly selected sample households comprising 138,850 individual cases were administered a Computer and Internet Use Survey, which covers all fifty states and the District of Columbia. The data used to examine Internet use among school-age children consisted of 23,323 reported cases between the ages of 3 and 18 years of age.

### Analytic approach

A sample of 23,046 school-age children (ages 3 to 18 and were not parents themselves), were available for the analysis. Three different measures of children’s computer use were examined. Specifically, the CPS Internet Use Survey collected information on whether school-age children used: 1) a computing device at home, 2) the Internet at home and 3) the Internet at school. This information was used to form the following policy relevant questions examined below.

### Sample definition and analytic dataset

Two conditions guided the development of the analytic dataset. The first was the selection of children 3 to 18 years of age. Second to identify a household using the unique household identifier, QST_NUM that lists persons as residing in a household at the time the survey was conducted [22]. Using this indicator allowed us to identify adults listed as residing in the household for the November of 2019 CPS survey and develop average measures of computers and Internet use per adult in a household.

### Dependent variables used in the analysis

The following measures were chosen for analysis in this study. First, the study examines three endogenous (dependent) variables: 1) children’s use of computers at home (For the purposes of this analysis, computers were defined as desktop computers, laptops, or tablets. Smartphones were not included because they were not considered useful for most educational purposes). 2) children’s use of the Internet at home and 3) children’s use of the Internet at school. Examination of the three dependent variables, in Table 1, shows 55.1% and 63.9% of children reported using a computer and the Internet at home and 49.2% of children reported using the Internet at school. Significantly 28.0% of children reported they did not use the Internet at school or at home and another 22.8% reported using the Internet at home but not at school.

**Table 1:** Dependent variables: Children’s (3 to 18) use of technology.

Technology	Percent Using	Children (3–18)
Using computer at home	55.1%	23,046
Using Internet at home	63.9%	23,046
Using Internet at school	49.2%	23,046

### Independent variables used in the analysis

The independent variables used in the analysis include three sets of individual level and household level factors. First, the analysis includes measures of the demographic characteristics of school-age children, specifically their gender and race/ethnicity. Next, the study incorporated measures on the financial and social characteristics of students’ families/households, including family income and the citizenship status, marital status, employment status and educational level of the household reference person (hereafter referred to as the Householder).(Household: A household consists of all the persons who occupy a house, an apartment, or other group of rooms, or a room, which constitutes a housing unit. A group of rooms or a single room is regarded as a housing unit when it is occupied as separate living quarters; that is, when the occupants do not live with any other person in the structure, and when there is direct access from the outside or through a common hall [22] (pg. 4–3). The person designated as the householder is the "reference person" to whom the relationship of all other household members, if any, is recorded [22] (pg. 4–3). This person is listed as ‘person number 1’ in the Census survey for a household (http://www.census.gov/population/www/cps/ cpsdef.html) 359)). (The geographic location variable available in CPS, GTCBSASZ, was not included because the bivariate analysis revealed no difference in use across the U.S. at the level of aggregation available in the CPS data. Differences by geographic location may be observed at a more disaggregated level with detailed PII data. This is a topic for future research). Finally, three proxy measures of the computing resources and experience of the adults (household persons over 18) in students’ households, were developed: 1) the average number of different computing devices (i.e., desktop, laptop computer and tablet computers) used by all adults in a household (up to three devices per adult), 2) the proportion of adults in the household that use the Internet in the home (from 0 to 1.0) and 3) the average number of different locations adults in a household accessed the Internet outside the home (i.e., at work, at school, at a café, at a library, community center or other public space, at someone else home, at some other location, or while traveling between locations (up to seven per adult). (Adult use of the Internet in different locations outside the home can serve as a proxy for adults’ familiarity with Internet technology but also potentially as a proxy for the level of local Internet provider availability. Measurement of the latter factor, however, requires disaggregated local level Internet provider data not available for this analysis).

#### 1. Child background characteristics

The first set of exogenous/explanatory variables used in this analysis includes a dichotomous variable for the gender (1 = male, 2 = female) of each child in the study. The race and ethnicity of the child is classified into five categories, 1 = white, non-Hispanic, 2 = Black, non-Hispanic, 3 = Hispanic, 4 = Asian, and 5 = Other non-Hispanic, racial categories.

#### 2. Family income, and the labor force status, education, martial status and citizenship status of the householder

The second set of explanatory variables includes the martial status and the education level of the householder. The marital status of householders was grouped into five categories: 1.0 = married spouse present, 2.0 = separated/spouse absent, 3 = widowed, 4 = divorced, and 5 = never married. The educational level of the householder was coded into the ordinal categories, 1.0 = less than High School, 2.0 = High School diploma, 3.0 = some college, and 4.0 = college degree and higher. The labor force status of the householder is classified into three categories: 1 = in the labor force and employed (variable 9 in Table 7), 2 = In the labor force and not employed (variable 10 in Table 7), and 3 = Not in the labor force, as the reference category. The citizenship status of the householder was classified as 1 = Natural born in the U.S., 2 = Native born in Puerto Rico or other U.S. Island areas, 3 = Native born abroad of American parents, 4 = Foreign born U.S. citizen by naturalization, and 5 = Foreign born not a citizen of the U.S.

The total income reported by families represents a measure of family financial resources. The family income variable is coded as 1.0 = under $10,000 per year, 2.0 = $10–19,999 per year, 3.0 = $20–34,999 per year, 4.0 = $35–49,999 per year, 5.0 = $50–74,999 per year, 6.0 = $75–99,999 per year and 7.0 = $100,000 and above per year.

#### 3. Adult household computer resources and internet utilization

Details of the variable measures used in this paper include: 1) The average number of computers per person up to 18 years of age per household is coded as 0 = no computers in household, 1 = .01-.49, 2 = 0.5–0.99, 3 = 1.0–1.99, 4 = 2.0–2.99, and 5 = 3.0. The proportion of adults in the household reporting they used the Internet at home is coded as 0 = not adults, 1 = .01 to .99 adults, and 1 = all adults. The average number of Internet locations accessed per adult outside the home is coded as 0 = No household Internet access, 1 = .01 to .99 locations, 2 = 1.0 to 1.99 locations, 3 = 2.0 to 2.99 locations, 5 = 3.0 to 3.99 locations, 5 = 4.0 to 7.0 locations.

## Statistical approach

The analysis is broken into two segments. The first examines the bivariate relationships between computer and Internet use among school-age children at home and other various locations (primarily at school) against independent explanatory factors that are potential determinants of computer and Internet use. Tables 2–10 present the basic bivariate analysis.

**Table 2:** Children’s technology use by gender.

Percent using technology	Male	Female	Total
% using computer at home	54.4%	55.9%	55.1%
% using Internet at home	64.2%	63.6%	63.9%
% using Internet at school	48.8%	49.5%	49.2%
Total cases	11,870	11,176	23,046
	100.0%	100.0%	100.0%

Chi-Square = 5.851, df 1, p < .016

**Table 3:** Children’s technology use by race/ethnicity.

Percent using technology	White	Black	Nat. Amer. Alaska Nat.	Asian	Mixed-Race	Hispanic origin	Total
% using computer at home	59.6%	48.2%	41.0%	56.7%	59.1%	46.3%	55.1%
% using Internet at home	66.8%	59.2%	52.6%	61.1%	69.3%	59.1%	63.9%
% using Internet at school	51.3%	45.6%	43.7%	42.0%	49.8%	47.1%	49.2%
Total cases	13,397	2,445	437	1,125	836	4,806	23,046
	100.0%	100.0%	100.0%	100.0%	100.0%	100.0%	100.0%

Chi-Square = 204.885, df 1, p < .000

**Table 4:** Children’s technology use by family income.

Percent using technology	0–9999	10–19999	20–34999	35–49999	50–74999	75–99999	100,000 +	Total
% using computer at home	30.0%	41.9%	39.9%	48.3%	56.2%	59.1%	66.9%	55.1%
% using Internet at home	44.2%	54.6%	54.6%	60.7%	62.6%	68.4%	71.8%	63.9%
% using Internet at school	41.6%	44.8%	43.8%	44.6%	48.1%	50.4%	54.7%	49.2%
Total cases	966	1,387	3,024	2,762	4,024	3,166	7,717	23,046
	100.0%	100.0%	100.0%	100.0%	100.0%	100.0%	100.0%	100.0%

Chi-Square = 1098.366, df 1, p < .000

**Table 5:** Children’s technology use by householder education.

Percent using technology	LT HS	HS	Some college	College +	Total
% using computer at home	34.3%	45.2%	57.2%	66.0%	55.0%
% using Internet at home	49.7%	57.5%	65.2%	71.4%	63.9%
% using Internet at school	40.1%	44.9%	49.5%	54.3%	49.1%
Total	2,438	5,624	6,555	8,213	22,830
	100.0%	100.0%	100.0%	100.0%	100.0%

Chi-Square = 1050.114, df 1, p < .000

**Table 6:** Children’s technology use by householder marital status.

Percent using technology	Married spouse present	Separated spouse absent	Divorced	Widowed	Never	Total
% using computer at home	58.4%	44.9%	55.9%	48.1%	42.1%	55.0%
% using Internet at home	65.4%	57.9%	66.7%	60.8%	56.6%	63.9%
% using Internet at school	49.8%	48.5%	54.4%	45.7%	42.0%	49.1%
Total cases	15,536	1,152	2,512	551	3,079	22,830
	100.0%	100.0%	100.0%	100.0%	100.0%	100.0%

Chi-Square = 265.027, df 1, p < .000

**Table 7:** Children’s technology use by householder labor force participation.

Percent using technology	in military	in labor force	unemployed	not in labor force	Total cases
% using computer at home	71.3%	56.7%	49.1%	48.6%	55.1%
% using Internet at home	69.9%	65.7%	61.0%	57.0%	63.9%
% using Internet at school	55.6%	50.1%	51.4%	44.9%	49.2%
Total cases	216	1,7835	521	4,474	23,046
	100.0%	100.0%	100.0%	100.0%	100.0%

Chi-Square = 113.315, df 1, p < .000

**Table 8:** Children’s technology by householder citizenship.

Percent using technology	Native citizen	Native born—PR/ other Islands	Naturalized U.S. citizen	Not a U.S. citizen	Total
% using computer at home	56.5%	42.7%	55.2%	41.9%	55.0%
% using Internet at home	64.9%	58.8%	65.3%	54.1%	63.9%
% using Internet at school	49.9%	41.2%	47.7%	43.6%	49.1%
Total cases	18,560	131	2,017	2,122	22,830
	100.0%	100.0%	100.0%	100.0%	100.0%

Chi-Square = 125.538, df 1, p < .000

**Table 9:** By the average number of computers used by adult in the household.

Percent using technology	0	.01 -.49	.5 - .99	1.0–1.99	2.0–2.99	3.0	Total
% using computer at home	17.2%	38.0%	47.5%	65.4%	76.1%	80.9%	55.1%
% using Internet at home	34.7%	65.7%	65.8%	71.5%	77.1%	79.6%	63.9%
% using Internet at school	28.8%	48.1%	46.2%	55.3%	58.3%	62.4%	49.2%
Total	5,428	437	2244	8,351	5,148	1,438	23,046
	100.0%	100.0%	100.0%	100.0%	100.0%	100.0%	100.0%

Chi-Square = 4812.845, df 1, p < .000

**Table 10:** Children’s technology use by the average number of adults who use the Internet at home in a household (home adult household internet utilization).

Percent using technology	No Adults use the Internet at Home	An average of .01 to .99 Household Adults use the Internet at Home	All Adults in the Household use the Internet at Home	Total cases
% using computer at home	14.4%	44.9%	64.0%	55.1%
% using Internet at home	10.9%	51.2%	75.4%	63.9%
% using Internet at school	15.1%	35.4%	57.2%	49.2%
Total cases	3314	2123	17602	23039
	100.0%	100.0%	100.0%	100.0%

Chi-Square = 5152.506, df 1, p < .000

Following the bivariate analysis, the unique contributions of each of the explanatory variables on computer and Internet use among school-age children is then explored through the use of logistic regression analysis.

In the model adopted in this study, the dependent variable is the natural logarithm of the odds ratio, y, of a school-age child using a computer at home, the Internet at home or at school. Thus, y = p /1-p and,

$$Ln\;(y)=\alpha_{oi}+\beta_{i}X+\varepsilon_{i}$$

where, α is the intercept, β is the i coefficients for the i independent variables, X is the matrix of observations on the independent variables, and εi is the residual or error term (See [23] (chapter 5, pp. 79–108) or [24].

For both the bivariate and logistic analyses, the analysis proceeds with the presumed relative temporal order of the explanatory variables relative to the three dependent variables, computer and Internet use at home, and Internet use at school by school-age children.

## Bivariate analysis of students’ computer and Internet use

The bivariate analysis, presented in Tables 2–12, examines the relationship of children’s use of technology resources (i.e., use of a computer at home, the Internet at home and the Internet at school) by their demographic characteristics, family economic and social characteristics and household technology resources. Table 12 shows use of the Internet at home and at school by the age of the child. As Table 12 indicates, among those children who do not use the Internet at home, a significant and consistent portion do not use the Internet at school as well, regardless of the age of the child. The percentage of students aged 3–5, 6–11, 12–14, and 15–18 who do not use the Internet at school or at home ranges from 48%, 29%, 22%, and 16% respectively. Although these percentages decreases with increasing age of the child, it remains significant (16% or more) regardless of age. This suggests that, regardless of the age of the child, a significant proportion of those who do not use the Internet at home do not use it at school either. As expected, the percentage of those 3–5 years of age who do not use the Internet at school is highest (49%). This makes sense since these early years are the least computer intensive.

**Table 11:** Children’s technology use by the average number of different Internet locations (Up to 7) outside the home accessed by adults in the household (adult internet utilization outside the home).

Percent using technology	no locations	.01 to .99 locations	1.0 to 1.99 locations	2.0 to 2.99 locations	3.0 to 3.99 locations	4.0 to 7 locations	Total cases
% using computer at home	23.8%	47.1%	63.3%	68.9%	72.1%	77.5%	55.1%
% using Internet at home	28.9%	59.2%	74.0%	77.6%	78.5%	81.8%	63.9%
% using Internet at school	19.6%	45.6%	57.4%	58.3%	62.4%	68.0%	49.2%
Total cases	4231	5226	6060	3613	2287	1629	23046
	100.0%	100.0%	100.0%	100.0%	100.0%	100.0%	100.0%

Chi-Square = 2413.089, df 1, p < .000

**Table 12:** Use of the Internet at home and at school by age of children.

		Does not use internet at home		Uses internet at home		
Age of respondent		Does not use internet at school	Uses internet at school	Does not use internet at school	Uses internet at school	Total All Locations
3 to 5	Count	1,975	240	1,327	587	4,129
	%	47.8%	5.8%	32.1%	14.2%	100.0%
6 to 11	Count	2,561	989	1,810	3,346	8,706
	%	29.4%	11.4%	20.8%	38.4%	100.0%
12 to 14	Count	980	334	844	2,240	4,398
	%	22.3%	7.6%	19.2%	50.9%	100.0%
15 to 18	Count	943.00	292	1,277	3,301	5,813
	%	16.2%	5.0%	22.0%	56.8%	100.0%
Total	Count	6,459	1,855	5,258	9,474	23,046
	%	28.0%	8.0%	22.8%	41.1%	100.0%

Chi-Square = 2528.618, df 1, p < .001

Table 2 shows children’s technology use by Gender. As shown in Table 2, there are only small (less than 2%) differences between males and females’ use of computers and the Internet at home similarly small between males and females’ use of the Internet at school.

Table 3 examines technology use by the race and ethnicity of children. With regard to use of computing devices at home, differences between Whites, Asians and Mixed-Race are relatively small (59.6%, 56.7% and 59.1%, respectively). However, compared with White children, Black and Hispanic children reported nearly 20% less used a computer at home (48.2% and 46.3%, respectively) and only 41.0% of Native American children reported use of a computer at home. Internet use at home by race and ethnicity of children shows a pattern similar to computer use at home. Approximately two thirds of Whites, Asians and Mixed-race children (66.8%, 61.1% and 69.3%, respectively) use the Internet at home while fewer Black, Hispanic and Native American children’s households use it from home (59.2%, 59.1% and 52.6%, respectively). Finally, as noted, children in general show lower levels of Internet use at school compared to use at home, but differences across race and ethnicity of children still persist although more modestly, where 50.4% and 48.1% of White and Mixed-Race children reported using the Internet at school, compared with 42.0% and 43.7% of Asian and Native American children.

With regard to family income and the level of education of the householder, reported data show similar patterns as expected (Tables 4 and 5). Children’s use of a computer at home varies from a low of 30.0% of those households earning under $10,000 annual income to a high of 66.9% of those earning $100,000 or more per year. Similar to family income, children’s use of a computer at home varies strongly with householder education from a low of 34.3% of those householders with less than a high school diploma to a high of 66.0% of those householders with a college degree and higher.

Tables 4 and 5 also show that children’s use of the Internet at home varies directly with family income and householder education. Internet use from home varies from a low of 44.2% of those households earning under $10,000 annual income to a high of 71.8% of those earning in excess of $100,000 per year. The level of education of the householder is also directly related to children’s use of the Internet at home and varies from a low of 49.7% of those householders with less than a high school diploma to a high of 71.4% of those householders with a college degree and higher.

Children’s use of the Internet at school is also correlated (but less strongly) with family income and householder education. Internet use at school increases from a low of 41.6% of households earning under $10,000 per year, to a high of 54.7% among those households earning over $100,000 per year. Similarly, children’s use of the Internet at school increases directly with householder education from a low of 40.1% among those with less than a high school diploma, to a high of 54.3% among those with a college degree and higher.

Children’s use of a computer at home is related to the marital status of the householder (Table 6). Those households most likely to provide children with computer use come from married, spouse present households (58.4%) while those children least likely (42.1%) to have use come from never married households. Perhaps surprisingly, marital status is less strongly related to children’s use of the Internet at home. Households most likely to provide Internet use at home come from married, spouse present, and divorced households (65.4% and 66.7%, respectively) while those least likely to use it come from never married and separated, spouse absent households (56.6% and 57.9%, respectively). Relative to Internet use at school, householder marital status shows a somewhat weaker relationship with children using the Internet at school. However, only 42.0% of students from never married households, reported using the Internet at their school.

Table 7 examines the relationship between householder labor force participation and children’s use of technology. Children’s computer use at home varies from a high of 71.3% of those in the military to lows of 49.1% and 48.6%, respectively) for children where the householder is unemployed or not in the labor force. Children’s reported use of the Internet at home varies from69.9% and 65.7% in households where the householder is in the military or in labor force, respectfully, compared 61.0% and 57.0% where the householder is unemployed or not in the labor force. With regard to children’s Internet use at school, children from households where the householder is not in the labor force report the lowest level of Internet use at school (i.e., 44.9% compared to 55.6%, 51.4% and 50.1%, where the householder is in the military, unemployed or employed, respectively).

With regard to citizenship status (Table 8), computer use by children from Native-born U.S. citizens and Naturalized households reported the highest level of computer use at home (56.5%, and 55.2%, respectively). In contrast, children where the householder was Native born in Puerto Rico or Foreign born not a U.S. citizen reported substantially less computer use at home (42.7% and 41.9%, respectively). With regard to citizenship status of the householder, the results for Internet use at home were similar to those for computer use at home.

Table 9 examines the relationship between the average number of computers used by adults in the household (up to three per adult) to children’s computer use at home, to their Internet use at home and their Internet use at school. As expected, children’s computer use is strongly correlated with the number of computers used by adults in the household. Only 17.2% of children reported using a computer at home in households where no adults reported using a computer. In contrast, 80.9% of children reported using a computer at home in households where adults reported using three different types of computers.

Table 9 examines the relationship between the average number of computers used by adults in the household to children’s Internet use at home. Not surprisingly, children’s use of the Internet at home is strongly related to the average number of computers per adult in the household. As reported, use varies from a low of 34.7% (for those children in household with no computers per adult) to a high of 79.6% reporting use in households where adults reported having an average of three computers. Table 9 also shows that adult computing resources are strongly associated with children’s Internet use at school. Children’s reported use of the Internet at school varies from a low of 28.8% for children in households where no adults reported using a computer to a high of 62.4% in households where all adults reported using on average 3 different types of computers, This may indicate that those with considerable at home computing resources are much more likely to send their children to schools which also has such resources.

Table 10 examines the relationship between adult Internet use at home (as measured by the average number of adults who use Internet technology at home, from none to all (i.e., 0 to a high of 1 and children’s use of a computer and the Internet at home and at school. As expected, there is a strong positive association between adults’ use of the Internet at home and children’s at home computer and Internet use, Children’s use of a computer at home varies from a low of 14.4% of children where no adults in the household use the Internet at home to a high of 64.0% of children in households where all adults use the Internet at home. In addition, there is a very strong positive correlation between children’s Internet use at home and Internet use by adults in the household. Children’s Internet use at home rises sharply from a low of 10.9% (in households where not adults use the Internet at home) to a high of 75.4%, where all adults in the household use the Internet at home. Thus, both adult computer and Internet use at home is strongly associated with Internet use by children in their household.

Table 10 also shows that Internet use by adults in the household also has a strong positive correlation with children’s Internet use at school. The reported outcomes increase sharply from a low of 15.1% for children in households where no adult uses the Internet at home to a high of 57.2% for those children where all adults in the household use the Internet at home.

Similarly, there is a strong association between children’s Internet use at home and Internet use by adults outside the home. Children’s Internet use rises sharply from a low of 28.9% in those households where no adults use the Internet outside the home, to a high of 81.8% of children in those households where adults accessed the Internet on average at four or more locations outside the home.

Finally, as Table 11 shows, Internet use by adults outside the home also shows a strong positive correlation with children’s Internet use at school. The reported outcomes range in a sharp positive linear fashion from a low of 19.6% among households with no Internet use locations (not online) to a high of 68.0% for those with 4–7 locations

## Multivariate analysis of students’ computer and Internet use

We now assess the potential impact of each of the independent variables (examined in the bivariate analyses) on students’ use of computer and Internet technology, controlling for the effects of the other independent variables. We employ logistic regression analysis to estimate the unique effect of each independent variable on the odds that school-age students 1) use a computer at home, 2) can use the Internet at home or 3) can use the Internet at school.

We examine the effects of the independent variables on each of three dependent variables. For each analysis, the potential effects of the independent variables are assessed for each of the three sets of explanatory variables (Blocks 1–3). The blocks are (1) children’s demographic characteristics, (2) socioeconomic characteristics of students’ families and households, including householder marital status, education, labor force status and family income and (3) adult computing resources and Internet utilization in the home and at locations outside the home, the latter of which can serve as proxy for adult expertise in Information technology.

Each block of explanatory variables enters the analysis according to its temporal position relative to the dependent variables and the other independent variables. The independent effects of individual explanatory variables are first assessed at the temporal stage as they enter the analysis. Importantly, this enables researchers to examine the mediating effect of the succeeding blocks of explanatory variables on children’s computer and Internet use as they are subsequently introduced into the analysis.

### Children’s use of computers at home

Table 13 presents the results of the logistic regression analysis of factors affecting children’s computer use in their home. Six variables representing child demographic characteristics enter the logistic regression analysis in the first stage (block 1). These include dichotomous variables representing the gender of the child, the gender, and race/ethnicity (White, Hispanic, Mixed-Race, Asian, and Native American/Alaskan) status of the householder (variables 1–6). The reference category for race/ethnicity is Black and for gender is male.

**Table 13:** Logistic regression analysis of factors affecting children’s use of a computer at home.

Independent Variables	Block 1			Block 2			Block 3		
	B	Sig.	Exp(B)	B	Sig.	Exp(B)	B	Sig.	Exp(B)
Child Gender–Male reference category	-	-	-	-	-	-	-	-	-
1. Child Gender—Female	0.063	0.018	1.065	0.066	0.016	1.068	.075	.014	1.078
Child Race Black reference category	-	-	-	-	-	-	-	-	-
2. Child Race White	0.462	0.000	1.587	0.132	0.006	1.141	-.021	.699	.979
3. Child Hispanic	-0.076	0.129	0.927	0.004	0.946	1.004	-.041	.499	.960
4. Child Asian	0.340	0.000	1.405	-0.060	0.455	0.942	-.093	.303	.911
5. Child Nat Amer, AK Amer	-0.295	0.005	0.744	-0.295	0.007	0.745	-.165	.183	.848
6. Child Mixed-Race	0.435	0.000	1.546	0.184	0.029	1.201	-.075	.416	.928
7. Family Income				0.158	0.000	1.171	.084	.000	1.087
Householder not in household reference category				-	-	-	-	-	-
8. Householder in Labor Force				0.010	0.779	1.010	-.114	.005	.892
9. Householder Unemployed				0.127	0.190	1.136	.030	.783	1.030
10. Householder in military				1.432	0.000	4.187	.222	.244	1.249
Householder less then high school reference category				-	-	-	-	-	-
11. Householder Ed College plus				0.799	0.000	2.224	-.111	.091	.895
12. Householder Ed Some College				0.645	0.000	1.905	-.092	.139	.912
13. Householder Ed HS degree				0.276	0.000	1.318	-.070	.249	.933
Householder not a citizen				-	-	-	-	-	-
14.Householder Native citizen				0.107	0.056	1.113	-.091	.149	.913
15 Householder Naturalized citizen				0.183	0.006	1.201	.102	.174	1.107
16.Householder Native citizen Puerto Rico				-0.073	0.698	0.929	-.235	.258	.791
Householder not married				-	-	-	-	-	-
17. Householder married present				0.154	0.001	1.167	.146	.004	1.157
18. Householder separated or absent				0.032	0.652	1.033	.028	.725	1.029
19. Householder widowed				0.277	0.000	1.319	.259	.000	1.295
20. Householder divorced				0.133	0.170	1.142	.335	.002	1.398
No adults use a computer reference category							-	-	-
21. Av# Computers per Adult .01-.49							.666	.000	1.947
22. Av# Computers per Adult .50-.99							.967	.000	2.631
23. Av# Computers per Adult 1.0–1.99							1.556	.000	4.739
24. Av# Computers per Adult 2.0–3.0							2.007	.000	7.438
No Adults in household use the Internet at home ref cat.							-	-	-
25. Av # Adults who use the Internet at home .01 to .99							.695	.000	2.004
26. All Adults in the household use the Internet							.953	.000	2.593
No Adults use the Internet outside of home ref cat.							-	-	-
27. Av# Internet locs. outside home per adult .01- .99							.182	.001	1.200
28. Av# of Internet locs. outside home per adult 1.0–1.99							.466	.000	1.593
29. Av# of Internet locs. outside per adult 2.0–2.99							.610	.000	1.841
30. Av# of Internet locs. outside per adult 3.0–8.0							.696	.000	2.006
Constant	-0.102	0.016	0.903	-1.498	0.000	0.224	-2.573	.000	.076

In the basic model (block 1), the Exp (B) coefficient for the gender of the child (variable 1) shows that the odds of female school-age children using a computer in the home are 1.065 times (or 6.5%) greater than for male school-age children, controlling for the other demographic variables in the analysis.

In contrast to gender, the first stage logistic analysis shows that race is a strong predictor of student computer use. With Black students as the reference category, the analysis shows that the odds of a White child having at home computer use is 1.587 (or 58.7%) and the odds for Mixed-Race and Asian children are 54.6% and 40.5% higher than for a Black child, controlling for the other demographic variables in block 1. There is no statistical difference in computer use between Black and Hispanic children and the odds of Native American children’s use are 25.6% lower than for Blacks, controlling for the other demographic groups.

The socioeconomic characteristics of children’s families (variables 7–20 in Table 13) enter the analysis in Block 2. As in the bivariate analysis, these included family income, householder labor force and employment status, military status, the education level of the householder, and the citizenship and marital status of the householder. Family income is included as an ordinal variable coded to the same categories used in the bivariate tables. These categories represent a modest collapse of the ordinal family income variable provided in the November 2019 CPS data.

When the Block 2 variables enter the analysis, family income, householder education, and householder marital status all show statistically significant, and especially in the case of family income and householder education, strong effects on the odds of children using a computer at home. The entry of Block 2 variables into the analysis also significantly reduces racial and ethnic disparities in children’s use of a computer. The odds of White and Mixed-Race children using a computer at home falls to 14.1% (from 58.7%) and 0.20% (from 54.6%) respectively compared to their Black counterparts, and there is no longer a statistically significant difference between Asian and Black children with the addition of Block 2 variables. However, the addition of Block 2 socioeconomic characteristics does not significantly reduce disparities between the Black children reference group and Native American children. Finally, the effect of gender remains small but statistically significant with the inclusion of Block 2 variables in the analysis.

Adult computer resources and Internet use in the household (variables 21–30 in Table 13) are introduced into the analysis in Block 3. Adult computer resources in a household enter into the analysis as a set of dichotomous variables because of the strong nonlinear relationship between children’s and adult computer use. For each increase in the average number of computers possessed by adults in a household (compared to households where no adults report computer use), the odds that a child in the household will use a computer increases by 194.7% (households with an average of .01 to .49 adults who use a computer), 263.1% (for households with an average of .50 to .99 adults with access), 473.9% (for households with an average of 1.0 to 1.99 adults with access) and finally 743.8% (for households with an average of 2 or more adults who use it). This indicates that not only the presence of an adult who uses a computer but also the intensity of use by adults are strongly related to children’s computer use at home.

Internet use at home by adults in a household is also related to children’s computer use at home. Specifically, while controlling on all other independent variables, children in households where adults report accessing the Internet at home are significantly more likely to report accessing a computer than children in households where no adults report using the Internet at home. Thus, the odds of children in households where some adults (i.e., .01 and .99 adults) and all adults report using the Internet at home are 100.4% and 159.3% more likely to use a computer at home than in households where no adults report using the Internet at home.

Surprisingly, independent of adult use of the Internet at home and adult use of computers, adult Internet use outside the home is also related to children’s computer use. Specifically, controlling for the other independent variables in the analysis, for each increase in the average number of different outside locations per adult in a household (0–7) who accessed the Internet, there was a (20.0%, 59.3%, 84.1% and 100.6%, respectively) increase in the odds that children in the household will use a computer. Thus, higher levels of available technology and adult familiarity with computer and Internet technology appear to be positively associated with a child using a computer at home while also controlling for the socioeconomic characteristics of the household.

Importantly, once adult computer ownership and Internet use are entered into the analysis in Block 3, the impact of race and ethnicity for White, Hispanic, Asian, or Mixed-race children on computer use at home are statistically insignificant or show slightly negative effects compared to Black children.

### Children’s use of the Internet at home

Table 14 presents the results of the logistic stepwise regression analysis of children’s Internet use at home. The basic model (Block 1) shows no statistically significant difference between male and female school-age children using the Internet at home. In contrast, race and ethnicity show a strong relationship with children’s Internet use at home. Compared to Black children the odds of White and Mixed-Race children using the Internet at home are 38.5% and 55.4% higher than their Black counterparts, but Native American children report less use (23.5%), Finally, Hispanic and Asian children show no statically significant difference in Internet use compared to Black children.

**Table 14:** Logistic regression analysis of factors affecting children’s use of the Internet at home.

Independent Variables	Block 1			Block 2			Block 3		
	B	Sig.	Exp(B)	B	Sig.	Exp(B)	B	Sig.	Exp(B)
Child Gender–Male reference category	-	-	-	-	-	-	-	-	-
1. Child Gender—Female	-0.027	0.324	0.973	-0.028	0.308	0.972	-.033	.300	.968
Child Race Black reference category	-	-	-	-	-	-	-	-	-
2. Child Race White	0.325	0.000	1.385	0.105	0.031	1.110	-.038	.499	.963
3. Child Hispanic	-0.006	0.910	0.994	0.064	0.245	1.066	-.031	.621	.969
4. Child Asian	0.078	0.293	1.081	-0.232	0.004	0.793	-.268	.004	.765
5. Child Nat Amer, AK Amer	-0.268	0.010	0.765	-0.244	0.022	0.783	-.116	.351	.891
6. Child Mixed-Race	0.441	0.000	1.554	0.267	0.002	1.306	-.035	.715	.966
7. Family Income				0.124	0.000	1.132	.078	.000	1.081
Householder not in household reference category				-	-	-	-	-	-
8. Householder in Labor Force				0.141	0.000	1.152	.056	.176	1.058
9. Householder Unemployed				0.241	0.013	1.272	.176	.109	1.192
10. Householder in military				0.763	0.000	2.146	-.050	.790	.951
Householder less then high school reference category				-	-	-	-	-	-
11. Householder Ed College plus				0.550	0.000	1.733	-.130	.053	.878
12. Householder Ed Some College				0.414	0.000	1.513	-.142	.025	.868
13. Householder Ed HS degree				0.176	0.001	1.192	-.106	.082	.899
Householder not a citizen				-	-	-	-	-	-
14.Householder Native citizen				0.081	0.147	1.084	-.028	.662	.972
15 Householder Naturalized citizen				0.232	0.001	1.262	.241	.002	1.272
16.Householder Native citizen Puerto Rico				0.062	0.740	1.064	-.078	.711	.925
Householder not married				-	-	-	-	-	-
17. Householder married present				-0.015	0.737	0.985	.020	.698	1.020
18. Householder separated or absent				-0.011	0.878	0.989	.085	.305	1.089
19. Householder widowed				0.218	0.000	1.244	.258	.000	1.294
20. Householder divorced				0.143	0.144	1.153	.508	.000	1.662
No adults use a computer reference category							-	-	-
21. Av# Computers per Adult .01-.49							.625	.000	1.868
22. Av# Computers per Adult .50-.99							.369	.000	1.446
23. Av# Computers per Adult 1.0–1.99							.314	.000	1.369
24. Av# Computers per Adult 2.0–3.0							.420	.000	1.522
No Adults in household use the Internet at home ref cat.							-	-	-
25. Av # Adults who use the Internet at home .01 to .99							1.636	.000	5.136
26. All Adults in the household use the Internet							2.525	.000	12.487
No Adults use the Internet outside of home ref cat.							-	-	-
27. Av# Internet locs. outside home per adult .01- .99							.257	.000	1.293
28. Av# of Internet locs. outside home per adult 1.0–1.99							.659	.000	1.933
29. Av# of Internet locs. outside per adult 2.0–2.99							.778	.000	2.177
30. Av# of Internet locs. outside per adult 3.0–8.0							.809	.000	2.246
Constant	0.386	0.000	1.472	-0.673	0.000	0.510	-2.593	.000	.075

In block 2, the socioeconomic characteristics of children’s families enter the analysis. Among these variables, family income, householder education, and householder labor force participation all show statistically significant effects, and in the case of family income and householder education, strong effects on the odds of children using the Internet at home. The entry of these variables into the analysis also helps account for some of the disparities between Black children and their White and Mixed-Race counterparts, with the effect of disparities between Black children and White and Mixed-Race children falling to 11.0% and 30.6% respectively. The difference between Hispanic and Black children remained statistically insignificant. The inclusion of socioeconomic characteristics of children’s families into the analysis produced a slight negative comparison between Black and Asian children, with Asian children showing 20.7% lower odds of accessing the Internet at home. The disparity between Native American and Black children remained essentially the same.

Block 3 introduces the potential impact of adult technology resources and experience into the analysis. Specifically, we examine the effect of adults’ computer use in the home, adults Internet use at home, and adults Internet use outside the home on children’s use of the Internet at home. Both the average number of adults in a household using a computer and the average number of adults accessing the Internet at home show a positive and significant effect on children’s Internet use in their homes. Not surprising, the level of Internet use at home by adults is more strongly related to children’s use of the Internet. Specifically, the odds of children accessing the Internet at home are 513% and 1,248.7% greater in household where some adults (on average .01 to .99 adults) or all adults in a household use the Internet at home, while controlling for the socioeconomic characteristics of households, child demographics and other technology use by adults in the household.

Interestingly, Internet use by adults outside the home is also related to Internet use by children in the home. Specifically, controlling for the other independent variables in the analysis, for each increase in the average number of Internet locations outside the home accessed per adult in a household (0–7), there were a 29.3%, 93.3%, 117.7%, and 124.6%, respectively, increase in the odds that children in the household will use the Internet at home. Thus, higher levels of familiarity with computer and Internet technology appear to be positively associated with a child using the Internet, controlling for the socioeconomic characteristics of the household and for other adult technology resources and experience.

Importantly, once adult computer ownership and Internet use are entered into the analysis in Block 3, the impact of race and ethnicity for White, Hispanic, Asian, or Mixed-race children on at home Internet use are either statistically insignificant or show slightly positive effects compared to their Black counterparts. The one exception remains Native American children. Here the disparity between Native American and Black children is reduced but still statistically significant.

### Children’s use of the Internet at school

Finally, as Table 15 presents, we assess the potential impact of each independent variable on students’ using the Internet at school. In the basic model (block 1), the gender of the children has no statistically significant effect on the Internet use at school. As in the previous two analyses, although somewhat weaker, the first stage logistic analysis shows that race effects whether a student uses the Internet at school, specifically the odds of White and Mixed-Race children using the Internet at school are 1.259 (or 25.9% higher) and 18.1% higher than Black children, controlling for the other student demographic variables in block 1. Hispanic and Native American children show no statistically significant difference with Black children in their use of the Internet at school, while Asian children show slightly lower odds of using the Internet at school.

**Table 15:** Logistic regression analysis of factors affecting children’s use of the Internet at school.

Independent Variables	Block 1			Block 2			Block 3		
	B	Sig.	Exp(B)	B	Sig.	Exp(B)	B	Sig.	Exp(B)
Child Gender–Male reference category	-	-	-	-	-	-	-	-	-
1. Child Gender—Female	0.029	0.273	1.029	0.030	0.266	1.030	.029	.312	1.029
Child Race Black reference category	-	-	-	-	-	-	-	-	-
2. Child Race White	0.230	0.000	1.259	0.082	0.080	1.086	-.027	.597	.974
3. Child Hispanic	0.062	0.216	1.064	0.108	0.041	1.115	.047	.411	1.048
4. Child Asian	-0.147	0.043	0.863	-0.327	0.000	0.721	-.362	.000	.696
5. Child Nat Amer, AK Amer	-0.075	0.471	0.927	-0.071	0.503	0.931	.065	.573	1.067
6. Child Mixed-Race	0.167	0.038	1.181	0.050	0.538	1.051	-.168	.049	.846
7. Family Income				0.060	0.000	1.062	.009	.372	1.009
Householder not in household reference category				-	-	-	-	-	-
8. Householder in Labor Force				0.073	0.038	1.076	-.043	.258	.958
9. Householder Unemployed				0.317	0.001	1.373	.300	.003	1.350
10. Householder in military				0.705	0.000	2.025	.039	.818	1.039
Householder less then high school reference category				-	-	-	-	-	-
11. Householder Ed College plus				0.423	0.000	1.526	-.139	.024	.871
12. Householder Ed Some College				0.290	0.000	1.337	-.174	.003	.840
13. Householder Ed HS degree				0.152	0.003	1.165	-.060	.290	.942
Householder not a citizen reference category				-	-	-	-	-	-
14.Householder Native citizen				0.016	0.776	1.016	-.091	.122	.913
15 Householder Naturalized citizen				0.029	0.651	1.030	-.008	.908	.992
16.Householder Native citizen Puerto Rico				-0.208	0.263	0.812	-.358	.068	.699
Householder not married reference category				-	-	-	-	-	-
17. Householder married present				0.111	0.014	1.117	.153	.001	1.165
18. Householder separated or absent				0.232	0.001	1.261	.310	.000	1.363
19. Householder widowed				0.383	0.000	1.466	.413	.000	1.511
20. Householder divorced				0.139	0.143	1.149	.366	.000	1.442
No adults use a computer reference category							-	-	-
21. Av# Computers per Adult .01-.49							.378	.000	1.460
22. Av# Computers per Adult .50-.99							.147	.013	1.158
23. Av# Computers per Adult 1.0–1.99							.289	.000	1.336
24. Av# Computers per Adult 2.0–3.0							.294	.000	1.342
No Adults in household use the Internet at home ref cat.							-	-	-
25. Av # Adults who use the Internet at home .01 to .99							.468	.000	1.597
26. All Adults in the household use the Internet (1.0)							1.168	.000	3.215
No Adults use the Internet outside of home ref cat.							-	-	-
27. Av# Internet locs. outside home per adult .01- .99							.751	.000	2.119
28. Av# of Internet locs. outside home per adult 1.0–1.99							1.091	.000	2.977
29. Av# of Internet locs. outside per adult 2.0–2.99							1.088	.000	2.968
30. Av# of Internet locs. outside per adult 3.0–8.0							1.326	.000	3.765
Constant	-0.192	0.000	0.825	-.893	0.000	0.409	-2.077	.000	.125

As in the previous analyses, Block 2 introduces socioeconomic characteristics of children’s families into the analysis. Among these factors, family income, householder education, householder labor force, citizenship and marital status all show statistically significant effects on use of the Internet at school. With the entry of socioeconomic characteristics into the analysis White, Hispanic, Mixed-Race and Native American children do not show a statistically difference in Internet use at school from their Black counterparts. However, with the entry of sociodemographic factors into the analysis the odds of Asian children using the Internet at school are now 27.9% lower than their Black counterparts.

Perhaps surprisingly, adult use of technology resources is related to the likelihood of children in their households using the Internet in their schools, controlling on children’s demographic attributes and family socioeconomic factors. As in the prior analyses, household computer resources and Internet use of adults in and out of the household are introduced into the analysis in Block 3. Looking first at adult computer use in the household, children are more likely to have Internet use at school in households where adults report using computers themselves versus households where no adults report using any computer. This suggests that households where, at least, some adult use computers at home are associated with a greater likelihood of children using the Internet at school, controlling on other demographic, socioeconomic and technology factors.

Internet use by adults in a household is also related to children’s Internet use at school. Specifically, compared to households where no adults use the Internet at home, the odds of children accessing the Internet at school, are 59.7% greater and 221.5% greater in households where some or all adults have access. In addition, controlling for the other independent variables in the analysis (including Internet use by adults at home) Internet use outside the home is also associated with the likelihood of children using the Internet at school. Specifically, compared to children from households where no adults reported Internet use at locations outside the home, for each increase in the average number of Internet locations accessed by household adults outside the home (i.e., .01- .99, 1.0–1.99, 2.0–2.99, and 3.0–7.0), there was a two to three times increase in the odds that children in the household will use the Internet at school. Thus, higher levels of familiarity with computer and Internet technology appear to be positively associated with a child using the Internet at school, controlling for the socioeconomic characteristics of the household and or the number of computers adults in household have themselves.

Importantly, once adult computer ownership and Internet use are entered into the analysis in Block 3, the impact of race and ethnicity for White, Hispanic, Asian, or Mixed-Race children on Internet use at school are either statistically insignificant or show only slightly positive effects compared with their Black counterparts. Even the effects on Native American children become slightly positive. Controlling for each of the other independent variables in blocks 1–3, the odds of Native American children using the Internet at school improve to a modest 6.7% higher than their Black counterparts.

## Key findings

- Overall, 55.1% of all school-age children (aged 3 to 18) reported using one or more types of computing devices (i.e., a desktop, laptop or notebook computer) at home, 63.9% of children reported using the Internet at home (some possibly on a mobile device), and 49.2% of children reported using the Internet at school. Significantly, 28.0% of children reported they did not use the Internet at school or at home and another 22.8% reported using the Internet at home but not at school.
- In terms of child demographics, children’s computer and Internet use at home or the Internet at school is largely independent of the gender of the child. In contrast, children’s use of computers and the Internet at home and the Internet at school is related to the race and ethnicity of the child. The first stage (Block 1) multivariate analysis found that that computer use at home was significantly higher for White, Asian, and Mixed-Race children than for Black Hispanic and Native American children. Similarly, the analysis found that Internet use at home was significantly higher for White and Mixed-Race children than for Black, Hispanic or Native American children. Race and ethnic disparities, although more modest, were also found regarding children’s access to the Internet at school.
- The effect of racial and ethnicity on children’s use of computing and Internet technology are substantially reduced but do not disappear when family resources and characteristics are introduced into the multivariate analysis in Block 2, and largely disappear when the computing and Internet resources and expertise of adults in households enter the analysis in Block 3. The one exception to this pattern is Asian children who have lower levels of computer and Internet use controlling on the variables in Block 2 and 3.
- The entry of family resources and characteristics into the analysis in Block 2 found that family income and the education of the householder had substantial positive effects on children’s computer and Internet use at home, and that the labor force, citizenship and marital status of householders had a modest relationship with children’s use of computers and the Internet at home. Interestingly, children in homes with the householder in the military showed higher levels of computer and Internet use at home than other labor force status categories. Finally, with the entry of Block 2 factors into the analysis, family income, and the education, labor status, marital status and citizenship status of the householders show modest associations with children’s Internet use at school.
- The entry of computing resources and experience into the analysis in Block 3 largely mitigates the effects of the race and ethnicity of children’s use of computers and the Internet at home and the Internet at school. The exception is Asian children who show a lower chance of using computer and Internet technologies, controlling for Block 2 and 3 factors.
- After measures of adults in a household use of technology in and out of the home enter the analysis in Block 3, some family and householder factors continue to have a significant but reduced associations with children’s use of computer and Internet technology Family income continues to have a modest but reduced effect on children’s use of a computer and the Internet at home, but not their use of the Internet at school. Marital status of the householder, (in particular divorced and widowed household heads) continue to show a positive association with children’s use of technology, the effects of householder labor force and citizenship status on children’s use of technology remain largely unchanged with the introduction of Block 3 variables.
- Equally important, the analysis also shows that the significant sociodemographic disparities in children’s use of computers and the Internet are largely mitigated by the use of computer and Internet technology by adults in the family/household. Of those households where adults without at home computing resources or Internet connectivity, children are forced to rely on access and use of these resources at school or at some other available location.
- The multivariate analyses found that children’s use of a computer and the Internet at home and the Internet at school varied significantly with the average number of computers used by adults in the household, adult household internet utilization in the home and by adult internet utilization outside the home controlling for each of the other independent variables.

## Conclusions

This study serves to highlight a number of important policy issues with regard to computer and Internet use and K-12 education in U.S society. The outcomes of this empirical study highlight a persistent underlying serious socioeconomic problem, namely that of a deepening societal break between those with resources and means to succeed and those without resources or assistance, i.e., those who are left behind.

This study serves as a pre-pandemic reference point for measuring the current state and future of educational technology available for all K-12 students in the U.S. Although other research is focused on Internet use at the household level [25], this study was focused on Internet use of the child at the household level. Our analysis of existing disparities reveals that many students lack access to the computing and Internet resources and expertise (at home and school) required for their educational preparation and for their entry into today’s workforce.

Importantly, this study looks at a critical early stage in today’s educational process. Specifically, to what extent do students access and use computers and the Internet in their homes and at school. In the Educational Technology Value Chain model [10], this is an early critical stage since without access to necessary educational technology resources, the ability of students and school systems to optimize educational outcomes and achieve success in subsequent stages of the model process, may be seriously constrained or unlikely.

Higher levels of adult Internet use are most likely associated with better household access to Internet availability and resources but also with adults being better able and willing to support and manage Internet use of children in their households. Unfortunately, these findings also indicate that those with no at home Internet use are also less likely to be able to use the Internet at their schools as well. This may indicate that these schools do not have access to educational technology resources needed due to fiscal constraints and/or perhaps limited broadband provider availability in their local

This suggests that the U.S. cannot continue to ignore these deficiencies and disparities but needs to consider investing more fully in educational technology in schools on a more comprehensive scale across all economic and demographic groups in order to optimize educational outcomes for all students and the economy.

Importantly, societal returns on such investments in school based educational technology could potentially be very great. Potential immediate benefits include quickly helping students acquire early foundational reading, writing and math skills that are critical to later educational success, improving the general academic performance of all K-12 students, and increasing student proficiency in STEM (advanced science and mathematics) and other key subject areas. Overall, investments in educational technology along with increasing expertise in delivering such technology in the short run, could help reduce the educational achievement gap, and over the longer term, help future Americans acquire the workforce skills necessary to be proficient, productive and innovative to help retain our technological and economic competitive edge in the global marketplace.

The findings indicate that a significant proportion of children in the United States do not have adequate access to or use education technology resources or to adult support and guidance in using these resources. This is not surprising given that many families may not have the financial means to acquire computing and Internet resources for their children. In addition, adults in many families may have limited time and/or the technical knowledge to help their children optimize the use of computers and the Internet for their education, and some families with these resources report difficulties monitoring their children’s use of these technologies [21]. As a result, at least for many families, schools function as a needed bridge to provide their children with the computers and the Internet resources and support required to optimize their education. Importantly, the analysis indicates that at least some sharp disparities in children’s access to educational technology can be associated with family income, education and race which could be significantly mitigated with more comprehensive programs in schools.

This study clearly shows that there are severe consequences to society for those without computing and Internet resources in their homes and at school. These gaps are not evenly distributed across society and will exacerbate existing digital and educational disparities in the nation. Many systematic and chronic underlying societal problems will be exacerbated and perhaps magnified as a result. In order to effectively mitigate the effects of limited educational technology resources and associated underlying societal impacts, and leverage the increasing benefits from an ever evolving educational technology value chain, we as a nation must commit more fully to providing incentives to help reduce this gap.

### Areas of future study

The present study is the first phase in our examination of the impact of household adult knowledge and familiarity with computers and the Internet on the ability of youth in their households to access this technology. The second phase will examine the impact of neighborhood social and economic resource availability by location on children’s use of computers and the internet, in addition to the familiarity with technology of adults in children’s’ households.

In this second phase, we will analyze children’s use of technology by the age of the child combined with their geographic location, in order to determine the use of educational technology in educational settings by academic age. In addition, we also will extend our exploration into the variation in school computing and Internet access resources among school age children by resource disparities in schools and school districts, within a community context.

**Project Data Sets found at:** https://ntia.gov/page/download-ntia-internet-use-survey-datasets
